# SERUM TRANSTHYRETIN LEVEL IS ASSOCIATED WITH COGNITIVE FUNCTION IN COMMUNITY-DWELLING OLDER ADULTS

**DOI:** 10.1093/geroni/igad104.3587

**Published:** 2023-12-21

**Authors:** Misa Nakamura, Masakazu Imaoka, Fumie Tazaki, Mitsumasa Hida, Ryota Imai, Takanari Kubo, Keiko Sakai, Masatoshi Takeda

**Affiliations:** Osaka Kawasaki Rehabilitation University, Kaizuka, Osaka, Japan; Osaka Kawasaki Rehabilitation University, Kaizuka, Osaka, Japan; Osaka Kawasaki Rehabilitation University, Kaizuka, Osaka, Japan; Osaka Kawasaki Rehabilitation University, Kaizuka, Osaka, Japan; Osaka Kawasaki Rehabilitation University, Kaizuka, Osaka, Japan; Osaka Kawasaki Rehabilitation University, Kaizuka, Osaka, Japan; Osaka Kawasaki Rehabilitation University, Kaizuka, Osaka, Japan; Osaka Kawasaki Rehabilitation University, Kaizuka, Osaka, Japan

## Abstract

Serum transthyretin (TTR) level has been shown in several studies to be associated with mild cognitive impairment (MCI) and dementia. This study aims to clarify the usefulness of TTR as a biomarker of change in cognitive function in people with normal cognitive function (NC) as a phenotype. We therefore investigate the relationship between cognitive scores and TTR in a cross-sectional study. A longitudinal study then examines the involvement of TTR level in the transition from NC to MCI. Cognitive function was evaluated using Addenbrooke’s Cognitive Examination-Revised (ACE-R). A cross-sectional study was conducted in community-dwelling older people (n=211) classified into NC, MCI, or dementia by ACE-R score. A 32-month longitudinal study was then conducted (n=29). Multiple regression analysis used sex, age, years of education and TTR as dependent variables and ACE-R scores as an independent variable. TTR was significantly associated with ACE-R in people with NC (β = -0.295, p =0.0008). No association with TTR was found in the MCI or dementia groups. Multiple regression analysis using sex, age and each ACE-R subdomain as dependent variables and TTR level as independent variable showed significant association with memory and language. In the longitudinal study, data analysis revealed that TTR level at baseline in females with MCI was significantly higher than it in females with NC. Our results suggest serum TTR levels might be a useful marker for cognitive function in preclinical AD. Elevated TTR might be a predictor of transition to MCI in old-aged adults with NC.

